# Barriers and facilitators to the utilization of cancer screening services in two Arctic Indigenous communities in Canada

**DOI:** 10.1080/16549716.2026.2657081

**Published:** 2026-04-20

**Authors:** Fariba Kolahdooz, Se Lim Jang, Kyoung June Yi, Sarah Deck, Crystal Milligan, Debbie DeLancey, Stephanie Irlbacher-Fox, Leah Seaman, Ryan Falk, Lianne Mantla-Look, Sarah Cook, André Corriveau, Carolyn Gotay, Kami Kandola, Sangita Sharma

**Affiliations:** a Indigenous Global Health Research Group, Department of Medicine, College of Health Sciences, University of Alberta, Edmonton, AB, Canada; b Department of Kinesiology, Faculty of Science, McMaster University, Hamilton, ON, Canada; c Dalla Lana School of Public Health, University of Toronto, Toronto, ON, Canada; d Aurora College, Yellowknife, NT, Canada; e Hotıì ts’eeda Northwest Territories SPOR Support Unit, Yellowknife, NT, Canada; f Beaufort Delta Region, Northwest Territories Health and Social Services Authority, Inuvik, NT, Canada; g Northwest Territories Health and Social Services Authority, Yellowknife, NT, Canada; h Independent Public Health Consultant, Yellowknife, NT, Canada; i School of Population and Public Health, University of British Columbia, Vancouver, BC, Canada; j Department of Health and Social Services, Government of Northwest Territories, Yellowknife, NT, Canada

**Keywords:** Indigenous, qualitative, remote communities, cancer screening services, Northern Canada

## Abstract

**Background:**

Indigenous communities in Canada have high age-standardized rates of cancer mortality. Remote communities in Canada’s northern territories have a high proportion of Indigenous residents and disproportionately low utilization rates of screening services for breast, cervical, and colorectal cancers, which could contribute to delayed cancer diagnosis and less favourable clinical outcomes. Knowledge is limited regarding the under-utilization of cancer screening services.

**Objective:**

This study identified factors contributing to the underutilization of breast, cervical, and colorectal cancer screening services and documented suggestions to promote utilization in remote Indigenous communities in Northwest Territories, Canada.

**Methods:**

This qualitative study consisted of four sessions of sharing circles and two one-on-one interviews with nine healthcare professionals, eight community members including Elders, and five community leadership from two Northwest Territories communities. Data were transcribed verbatim and coded and analyzed using NVivo-10. The constant comparative method determined emergent themes.

**Results:**

Data identified four themes of factors influencing the utilization of cancer screening services (socio-environmental, socio-cultural, socio-political, and personal themes), covering limited resources, limited cultural considerations, the legacy of colonial practices, and fear. Five themes regarding suggested approaches to encourage utilization were community outreach, collaboration, sustainability, cultural safety, and support for healthcare professionals.

**Conclusions:**

Factors identified to affect cancer screening service utilization and suggested approaches to improve the utilization are supported by other studies and initiatives in the region highlighting the relevance and feasibility of the findings. The findings could further inform community-based interventions for improving the utilization of cancer screening and support services in remote Indigenous communities.

## Paper Context

- **Main findings:** This qualitative study explores socio-environmental, socio-cultural, socio-political, and personal aspects of utilizing publicly funded breast, cervical, and colorectal cancer screening services in remote Indigenous communities in Northwest Territories, Canada.
- **Added knowledge:** Addressing low rates of utilizing cancer screening services in remote Indigenous communities in Northwest Territories requires implementing community outreach, encouraging community collaboration, supporting sustainability, establishing cultural safety, and providing support for healthcare professionals.
- **Global health impact for policy and action:** The findings can inform interventions, practices, and policies to improve the utilization of cancer screening services for remote Indigenous communities, where increasing cancer mortality warrants promoting the utilization of cancer screening services and early detection.

## Background

Cancer is the second leading cause of death worldwide; annually, approximately 10 million deaths are attributed to the disease [1]. In Canada, cancer is the most prevalent cause of death accounting for one in four deaths; it was projected that 85,000 Canadians would die from cancer in 2021 [2]. Colorectal cancer in males and breast cancer in females are second to lung cancer in both sexes as the most common causes of cancer mortality in Canada [2]. In 2021, the Arctic region of Canada, specifically Northwest Territories, Yukon, and Nunavut, had the highest age-standardized cancer mortality rates in the country, reporting 189.6, 205.0, and 224.5 per 100,000 deaths, respectively, compared to the national rate of 185.9 per 100,000 deaths [2].

Diagnosing cancer early through routine screening is essential to the reduction of cancer mortality rates. Routine cancer screenings can prevent premature deaths from cancer; breast cancer screening can reduce mortality rates for women aged between 40 and 84 years by approximately 40% [3], colorectal cancer screening reduces mortality rates by 67% [4], and cervical cancer screening reduces mortality rates by approximately 80% [5]. The Government of Northwest Territories’ cancer screening guidelines recommend the fecal immunochemical test (FIT) every 1 to 2 years for individuals ages 50 to 74 with an ‘average risk’ for colorectal cancer [6]. For women with an ‘average risk’ for breast and cervical cancers, the guidelines recommend a mammogram every 2 years from ages 50 to 74 [7], and the Papanicolaou test (Pap smear) every 2 years following three annual tests with ‘normal’ results, starting 3 years after becoming sexually active or at 21 years old [8].

Cancer screening utilization rates in Northwest Territories are among the lowest in Canada [9]. Only 20% of eligible Northwest Territories residents had a screening test for colorectal cancer in 2011–12 [10], a rate which increased to 25% in 2013–14 [11]. By contrast, in 2012, Manitoba and Ontario achieved routine screening participation rates of 36.1% and 55.6%, respectively [12]. In Northwest Territories, screening rates drop further within small remote communities. In the most recent Northwest Territories data, Yellowknife (the capital city) reported screening rates for colorectal, breast, and cervical cancer as 32%, 55%, and 57%, respectively, whereas remote communities reported rates of 16%, 54%, and 47%, respectively [6–8]. Following a recent pilot programme to increase the rates of colorectal screenings using the FIT, the territorial utilization rate was still only 22%, far from the national target of 60% [13].

To date, there are limited data on what factors contribute to low rates of screening service utilization. The remoteness and small size of many Northwest Territories communities potentially reduce the amount of available healthcare professionals and necessary healthcare equipment. To access and utilize screening services, residents may need to travel to larger centres offering advanced healthcare services. These barriers could negatively impact individuals, increasing the time and finances required for screening [14]. Limited health literacy and body shyness, particularly regarding cervical cancer among First Nations women, were reported as factors hindering screening utilization [15], but there is currently no such information on factors affecting screening utilization specific to Indigenous communities in the Canadian Arctic. Cultural factors may also affect screening utilization rates. In the majority of Northwest Territories communities with fewer than 1,000 residents, 82.9% to 100% of residents are Indigenous (First Nations, Inuit, or Métis) [16]. Indigenous communities hold unique perspectives on cancer and preferences towards traditional healing practices [17]. Culturally safe interventions providing information that accounts for cultures and traditions could improve knowledge of cancer screening, enable informed decision-making, and increase screening utilization rates.

The ACCESS project (**A**ttitudes towards **C**ancer in Indigenous **C**ommunities & **E**xamining utilization of cancer **S**creening **S**ervices) aimed to explore Indigenous community members’ perceptions towards and experiences of cancer screening services in two Northwest Territories communities. This study explored factors impacting screening service utilization from the perspectives of key community informants. Two main questions guided the research: what factors affect the utilization of cancer screening services in Northwest Territories and what approaches can improve cancer screening utilization?

## Methods

### Setting

The study was conducted in two Northwest Territories communities. Community A is a small town with approximately 3,400 residents, 70% of whom self-identify as Indigenous. The sole hospital in the community has a permanent staff of doctors and nurses and is equipped to provide breast, colorectal, and cervical cancer screenings. This community also has one public health centre, and the town is accessible via road 9 months of the year.

Community B has approximately 550 residents, 90% of whom self-identifying as Indigenous. This community is isolated, accessible only by air year-round and by boat in the summer. Ice road access is limited to a few months during the winter. The health centre is staffed with a few permanent nurses, and a doctor visits monthly by air throughout the year. Pap smear and FIT services are provided by the health centre. Women travel to a larger community to receive mammography screening and follow-up.

### Design and recruitment

A range of healthcare professionals who delivered healthcare services including the cancer screening programmes at the local health centre, Indigenous leaders who were involved in community programmes and policies, and community members including Elders, cancer survivors, and volunteers of a local group promoting cancer prevention were approached in person and purposefully recruited using a convenience method to explore perspectives of individuals with expertise, knowledge, and experiences regarding cancer screening services. A maximum variation recruitment strategy was used to enhance the transferability of findings. Considering the interconnectedness of people in remote communities, a snowballing strategy (recruiting participants on the recommendation of individuals who had already participated in the study) was also used [18].

### Data collection

Discussions were conducted utilizing *sharing circles*, a culturally appropriate methodology that employs local Indigenous protocol to gather and share knowledge about specific topics while sitting in a circle [19]. During the sharing circles, participants engaged in open discussions regarding perceptions of cancer screening within the community [20,21]. Following a community-based participatory research approach, the discussion guide was developed in consultation with the Community Advisory Board (made up of 25 partner organizations, community members, and Elders) and included three questions: what factors might have positive influences on the utilization of cancer screening services in your community, what factors might have negative influences on the utilization of cancer screening services in your community, and what can be done to promote the utilization of cancer screening services in your community? These questions provided a framework, but participants guided the discussions and presented information that was not anticipated [22].

A non-Indigenous female author (SLJ) with a bachelor’s degree was the project lead and moderated the four sharing circles (two gatherings per community, with three to six participants in each session) in English with a research assistant as a notetaker. Before visiting the communities, the moderator received a training on qualitative methodologies, data collection, and interpretation. The moderator and the research assistant were ‘outsiders’ to the communities but were guided by the Community Advisory Board to mitigate cultural bias. The author and the research assistant stayed in the community to build rapport with community members and conduct research activities. It was obvious for community members that the author and research assistant were visiting from outside the community and they explained the purpose of the visit and the project. Two sessions in each community were held in the office, community health centre, or community drop-in centre that was reserved for the sharing circle; in Community A, one was with the public health centre staff, and the other with an Indigenous organization, and in Community B, one was with the public health centre staff, and the other was a local group that promote cancer screening, consisting of Elders, cancer survivors, and volunteers. Each sharing circle lasted approximately 90–120 minutes in length, and data were recorded by notetaking as verbatim as possible with extra memos to note moderator’s and notetaker’s impressions and insights during the discussions. No audio or video recording took place to respect participants’ preferences. Two participants from another Indigenous organization in Community A, who expressed interest in but were unable to attend the sharing circles, were interviewed separately in one-on-one semi-structured interviews using the same discussion guide as the sharing circles. The sharing circles and interviews were closed, with only the moderator, notetaker, and participants being present in the space. Attendants of the four sharing circles and two one-on-one interviews completed a demographic survey including age, gender, ethnicity, and work sector prior to participating in the discussion. After each session, the moderator and research assistant debriefed the discussion and compiled the notes to produce transcripts. Transcripts were not returned to participants, but nine authors who are healthcare professionals and stakeholders with extensive experiences and insights regarding cancer care in Northwest Territories reviewed the analysis and the manuscript for accurate representation of the data. No repeat interviews were conducted.

### Ethical considerations

All procedures were performed in compliance with relevant laws and institutional guidelines. Ethical approval was obtained from the Research Ethics Board at the University of Alberta who issued the research ethics certificate on 26 May 2014 (Pro00044165). Following the Northwest Territories *Scientists Act*, researchers also obtained a research licence from the Aurora Research Institute. Before the interview, participants signed informed consent. Data were stored in separate locked filing cabinets, and all electronic data were de-identified and password protected.

### Data analysis

This study utilized a thematic analysis method. Data consisted of transcripts and notes taken by the moderator and research assistant during the sharing circles and interviews [18,23,24]. The moderator and research assistant cross-checked the transcripts for accuracy [25] and compiled the data into narrative form [23]. During data collection, the moderator and research assistant also wrote field notes to record the context (atmosphere) of the sharing circles and interviews and memos to note possible emerging themes [24]. Field notes were used as supplementary evidence to the discussion data [18]. Two authors (KJY and SLJ) engaged in data coding and thematic analysis by reading the data line-by-line, selecting central concepts by identifying recurring topics, organizing and clustering selected concepts into themes and sub-themes [18], and then discussing the categorization of themes and sub-themes, comparing notes and settling any disagreements. A third author (FK) offered additional insight on final categorizations. Together, the three authors completed the thematic analysis and produced the final themes and sub-themes. Data were analyzed using NVivo 10 (QSR International Pty Ltd, 2018). The data were consistent in pattern of themes and adding the last transcript in the analyses did not reveal new themes, suggesting data saturation.

## Results

### Participant demographics

A total of 22 individuals participated (Table 1). The majority (86%) identified as female, and 68% were Indigenous. Participants were almost evenly distributed into the age groups of 30–39, 40–50, and ≥51 years. Most participants worked in the public sector (77%), 9% in the governmental sector, 9% in the voluntary sector, and 5% in the private sector. With relation to cancer screening services, 41% spoke from a healthcare provision focus, 36% from a health promotion focus, and 23% from a community development focus.

**Table 1.** Self-identified sociodemographic characteristics of participants in two communities in Northwest Territories (*N* = 22).

Characteristics	n (%)
Age group (years) 30–39 40–50 51 and over	8 (37) 7 (32) 7 (32)
Gender Female Male	19 (86) 3 (14)
Ethnicity First Nations (Dene) Non-Indigenous Inuit (Inuvialuit) First Nations (Gwich’in)	7 (32) 7 (32) 6 (27) 2 (9)
Work sector Public Governmental Voluntary Private	17 (77) 2 (9) 2 (9) 1 (5)
Primary focus in relation to cancer screening services Healthcare Health promotion Community development/leadership	9 (41) 8 (36) 5 (23)

### Contextual themes of factors influencing the utilization of cancer screening services

Four themes categorized the reported factors influencing screening utilization: socio-environmental, socio-cultural, socio-political, and personal. These themes were further divided into sub-themes. See Table 2 for a summary of these contextual themes and selected quotes.

**Table 2.** Contextual themes influencing the utilization of cancer screening services in two communities in Northwest Territories.

Themes	Sub-themes	Quotes
Socio-environmental	● Limited and transient healthcare/health promotion professionals ● Ineffective communication systems among healthcare professionals and levels of the health system ● Limited presence or awareness of resources and information about cancer screening services	*‘Here, no permanent doctor. One doctor visits the community once a month, for a week at most. During this time, it is extremely difficult to find the time to see the doctor, as the doctor is usually booked solid … .If someone finds a lump, she waits until the doctor is in town, then has to wait until the next visit to have the test performed, and then the next visit to hear the results of the test.’* – Community nurse *‘Doctors are often “on” and then “off” to travel back home. If people are sick, they will wait three months to see that particular doctor, because they do not want to see a different doctor and repeat themselves … .Nurses are similar, they only come for a few months, and then leave … .people may have different nurses every few months.’* – Health centre staff *‘[Getting] a second opinion … . with a limited number of doctors available; people only get diagnosis from one doctor, who may have made a mistake.’* – Healthcare professional *‘Transient doctors- build trust with the doctors been around for a while. Then they’ll stay sick. Get the other doctor, but no they’ll just wait for the other doctor to come back’* [When some community members are sick, they will wait to go to the doctor until the doctor they know and trust is available or in town] – Healthcare professional ‘*Permanent doctors were hired for [community A] last year. The health authorities specifically targeted couples, in order to increase the chances of them staying in [community A] … both are doctors who negotiated with them to make [community A] their home.’* – Healthcare professional *‘There [are] no Electronic Medical Charts. Each healthcare centre has their own chart. Typically, you are not seeing the same doctor and your record can be everywhere, so the hospitals and public health centre have a lot of difficulty sharing patient information. Communication is a big barrier in continuity of care.’* – Community nurse *‘The small communities are put to the side [in planning and delivering screening services].’* – Healthcare professional *‘We now have a database for FIT and colorectal cancer screening … It’s a small town. This is very effective for us to know everyone who is due and when. Plus, we can track the history of families.’* – Community nurse ‘*You have to have items like posters for local people, people from the region. Hopefully they can relate to local people’* – Healthcare professional. ‘*I don’t think there is enough information out there.’* – Healthcare professional *‘Don’t usually see any information – like in the post office or Northmart, there is no information. If there was more information, I would see more.’-* Director of community organization
Socio-cultural	● Unfamiliarity with western medicine ● Unique Indigenous cultures ● Communication barriers between participants and healthcare professionals	*‘I think traditionally we never had to go for those tests. It’s not in our culture to do certain things … . we have believed nobody in our culture died of disease.’* – Community cancer group member *‘Instead of picking up pills at the drugstore, they will use Spruce Gum or Labrador Tea, Willows, and Juices. This is what our people did before all the pharmaceuticals came in.’* – Healthcare staff *‘When people have cancer they don’t know if they’re going to live. After they become a cancer survivor, they know they will live, and other people see that they can survive.’* – Health centre staff *‘People now see screening as preventive, rather than (as) diagnosis.’* – Community development practitioner *‘Many Elders think they are taking up doctors’ time and being a burden to them.’* – Indigenous health staff member *‘[Men] think it’s a sign of weakness. They just wait for cancer complication, saying “we are not asking help for minor ailments!”’* – Community cancer group member *‘[men] do not go for checkup, unless they are in so much pain. They go to hospital crawling.’* – Community development practitioner *‘I had trouble finding words for “rectum” or some “private areas.” I really didn’t want them to feel offended.’* – Volunteer language interpreter
Socio-political	● Continuing effects of residential schooling ● Uncongenial administration of health policies ● Limited support system, particularly for medical travel	‘[*Indigenous] people are taught to accept what the doctor says and not to ask questions. Especially, Elders will just listen to everything doctors say without asking any questions, even if they don’t understand. Nod and smile, nod and smile.’* – Health centre staff *‘When I went through the exams, nurses usually never told me anything. Just told me to wait at home. Then all of sudden, I was told “you are going to Yellowknife for an operation. You have cancer.” After that, I knew that I had to advocate for myself.’* – Community cancer group member *‘They [the governments and health authorities] only look at people who are in the high risk.’* - Participant *‘Travel [i.e. medical travel to a major city to receive further services] is minimum three days. It means three days away from work.’* – Community development practitioner *‘You have family, kids to take care of. If you gotta go to Edmonton, you gotta go. It could be the “scariest thing” for them.’* - Community development practitioner
Personal	● Fear and anxiety ● Emotional costs ● Negative past experiences in cancer screening services ● Limited self-reported importance of health-seeking behaviour	*‘[People] find that they want to be screened, because they are afraid that they might have cancer, and they want to know.’* – Health centre staff *‘Some people have fear to go for testing and find out they have cancer. Some may know there is something wrong, but don’t want to hear that they have to go through chemo[therapy].’* – Community nurse *‘It’s not walking to the doctor’s office. Here we are talking about someone who has to fly to Yellowknife or Edmonton [hours of flights] without knowing anything. It’s an untapped world for some of our people.’* – Health centre staff *‘A lot of times we’re given the diagnosis in Edmonton with nobody there. A few years ago, I had to go to Edmonton to get CAT [computed axial tomography] scan. After that, I was sitting there alone, thinking what if I would have cancer. Imagine you hear that you have cancer, and you are alone.’*- Community development practitioner *‘Wait to be called in before they go get their screening done.’* - Nurse *‘Some people, not all the people in the community, they put their health on the bottom of their list.’* – Healthcare professional

#### Theme 1: socio-environmental

Participants described characteristics of the physical and social setting as influential towards cancer screening utilization; these characteristics included the small sizes and remote locations of communities, limited access to and availability of services, and limited information on existing cancer screening services. Most participants also believed that the transiency and limited number of healthcare professionals negatively impacted cancer screening utilization.

Effective communication within the healthcare system was described as critical to the continuity of care in remote communities; inefficient information sharing and communication systems were noted as significant barriers to such care. One participant shared a perception that the territorial government overlooked smaller communities in the planning and implementation of screening services, thus limiting access. A nurse noted that the system of faxing referrals to larger centres was inefficient, with referrals for screenings taking up to a year, at times getting lost with no opportunity for feedback. One participant expressed that communication challenges could have consequences such as *‘[missed] surgeries and then months-long waits for treatment, or in the worst case, death.’* Some respondents suggested a locally established database system would be a positive change. A healthcare professional also suggested that the recruitment of permanent healthcare professionals, particularly couples, would be beneficial.

Most participants described existing informational materials, such as posters and pamphlets, and educational initiatives, such as visual demonstrations of screening procedures and community outreach programmes, as insufficient. Participants stressed limited awareness of these established resources, underlining a need for better promotion.

#### Theme 2: socio-cultural

Socio-cultural factors, such as the dominance of the western biomedical system, cultural views related to care-seeking, and communication barriers between clients and healthcare professionals were reported to affect cancer screening utilization.

Participants recognized that there has been an increased awareness of cancer and interest in related screening services. However, it was suggested that Indigenous cultural perspectives around care-seeking behaviours may still affect cancer screening utilization; several participants stated that a common belief among Indigenous community members is that *‘people should not burden others,’* and therefore individuals may not seek cancer screening, especially when asymptomatic. Participants also shared gendered perspectives regarding screening service utilization, specifically that men are less willing to risk potentially being perceived as weak by utilizing screening services.

The potential language barriers between Indigenous community members and non-Indigenous healthcare professionals were a topic discussed in detail. Potential barriers included the wide range of local Indigenous dialects within communities, having few translators, and the limited availability of appropriate resources to translate medical terminology. One participant, a volunteer interpreter, described how communication in medical settings becomes even more challenging when dealing with sensitive topics such as cancer.

#### Theme 3: socio-political

The combination of social and political factors, particularly the continuing impacts of residential schools, were identified as substantial barriers to screening utilization.

Participants described the acute impacts of racial discrimination and marginalization, and the subsequent impact of broken trust with the healthcare system. Some participants specifically referred to a culture of not questioning white authorities, originating from the legacy of residential schools, a forced assimilation policy and practice of the Canadian government from 1880 until 1996. During this time, Indigenous children were forcefully removed from families and communities and forced to live in residential schools, where practicing Indigenous cultures and traditions was forbidden. The personal and collective impacts of this government policy include a diminished sense of autonomy [26,27].

Several participants also reported that the healthcare system has focused on regional health issues and treatments other than cancer and cancer screening/prevention. Furthermore, although the federal and territorial governments fund certain medical travel expenses, there can be considerable financial burdens placed on individuals and families, such as funding childcare and missing work.

#### Theme 4: personal

Participants discussed personal factors affecting screening services utilization, including fear and personal decisions or attitudes against prioritizing health.

Fear was identified as both a motive and a deterrent for utilizing screening services. Community members may require medical travel to a major city for advanced screening, diagnosis, treatment, and follow-up care; medical travel was described as an ‘*anxiety-provoking process*’ and a significant barrier to pursuing screening services. Having to travel to a potentially unfamiliar city environment was also described as a deterrent to screening. Even more daunting was the prospect of travelling alone and potentially receiving bad news.

Participants also expressed that some community members may simply not be interested in screening services. Participants speculated that some community members, particularly men, may not feel the need for preventive screening if no symptoms are present; individuals may also find routine screening inconvenient, prioritizing other commitments. One Elder predicted such attitudes would be difficult to change, as they are *‘just a matter of personal choice.’*

### Community suggestions to promoting the utilization of cancer screening services

Participants recommended five approaches for improving cancer screening and utilization: community outreach, community collaboration, sustainability, cultural safety, and support for healthcare professionals. Participants also discussed practical suggestions for implementing these approaches. These recommendations are not distinctive; rather, they complement each other and can be implemented in concert. For example, the suggested local advocate could employ community outreach and community collaboration, contribute to sustainability and cultural safety, and support healthcare professionals, when promoting cancer screening services. These findings and selected quotes are summarized in Table 3.

**Table 3.** Community suggestions to promoting the utilization of cancer screening services and relevant quotes.

Suggestion		Quotes
Community outreach	● Reach out to the community through Indigenous community organizations ● Accommodate community needs and aspirations in service delivery ● Increase accessibility through mobile community outreach programmes ● Use existing community events for effective and efficient outreach	*‘I think the information is out there, but it’s just not getting to our people. They will pick up the information slowly, so you can’t just throw information at people all at once and expect them to take them up.’* – Community health promoter *‘The key is reaching out to our people: Reaching out to the small communities through Indigenous organizations, home visits for Elders … ’* – Community leader *‘[People] usually forget young people. Their family member may be suffering from cancer, but we hide them behind the door.’* – Community development practitioner *‘If young people are aware, if they learn about cancer as preventable, then they will share their knowledge with their parents and grandparents. We can also provide stats or fact sheets to bring home.’* – Community development practitioner *‘If [the] message doesn’t get repeated every year, it’s forgotten …. Reinforce all the time, otherwise it’s forgotten.’* – Health centre staff *‘Not just going into the communities and saying, “We’re doing this”, [and] show up at this time … If going into the communities, need to work around their time.’* – Community development practitioner *‘I saw in Manitoba, they had a van with a mammogram machine. The van comes down the winter road and does the cancer screening … This may be more needed in smaller communities.’* – Community cancer group member *‘We still have doctors here and a lab … . If we want to go for Pap, mammogram, colonoscopy, it’s all in town.’* – Community development worker *‘These events [traditional activities] are part of our life and a really good place and time to provide that information … . If you talk about cancer, doing things like hand games or sewing, people will listen.’* – Community development worker
Community collaboration	● Involve community organizations in service planning and delivery ● Promote community support ● Include community leadership groups within support system ● Work collaboratively among healthcare professionals and across local, territorial, and provincial jurisdictions ● Share medical information and expertise among healthcare professionals	*‘If the hospital was doing something, why not contact us? We can help make people more aware.’* – Director of a community organization *‘I think our health system needs to work with us to make sure we’re all on the same page. If the health system works with us, then our people will be more willing to say ok.’* – Community nurse *‘Not only us to work on this. Radio station, schools, Elders, store managers all need to focus on prevention and awareness. They are a huge influence in our life … . they need to know [prevention] will be a lot easier than helping cancer patients and their families.’* – Community cancer group member *‘A network is important. We need a team here and in Yellowknife and even in Edmonton to communicate who is coming for what and when. A person travelling for tests could directly contact that team as soon as they get to Yellowknife or Edmonton so that they can check up on the person and help the person settle in.’* – Community nurse *‘Diagnosis and after-care should go together. A lot of times people get lost where to go. We are only working for promotion, nurses are working for diagnosis, and someone else is focusing on treatment.’* – Health promoter
Sustainability	● Build long-term trusting relationships between healthcare professionals and clients ● Increase permanent healthcare professionals ● Educate and hire local people to be health advocates ● Promote ownership of health through education ● Increase awareness of cancer screening services through education ● Promote a culture of preventive behaviours through education ● Promote stories of early detection and its positive outcomes ● Educate younger generations through school educational systems ● Provide continual education and promotion	*‘Having a permanent family doctor would go a long way towards building trust. They can provide you with advice, and you would trust in them and having a long-term relationship with someone would go a long way towards getting people to use services they wouldn’t normally use.’* – Director of community organization *‘If we have permanent staff that stay in the community and know people in the community, it’s easier to follow up with cancer screening.’* - Nurse *‘[you] need to work around [community members] time, you need to do it in the evening, provide soup and bannock [then] people will come.’* – Community development worker *‘Utilize the radio [to promote] cancer screening.’* – Community development worker
Cultural safety	● Use community events for education and promotion ● Examine unique circumstances of living in remote community ● Examine complex root causes of lower utilization of the services ● Hire healthcare professionals who are familiar with Indigenous culture ● Use relatable images and understandable languages in promotion ● Provide diverse media and venues for promotional materials	*‘This is our way of healing, our way of sharing knowledge, our way of educating each other. In January’s sharing circle, we have talked about cancer, our own experiences and experiences of our friends and families with cancer. It was meant to be a safe place to express any feelings and thoughts.’ –* Community health promoter *‘These [cancer screening services] are something new to our culture. Once people become familiar with them, they will be more likely to do the tests. Our job is to encourage people to talk more about the tests.’* - Participant *‘They think our people are irresponsible for their own health.’* – Community health centre staff *‘They don’t care? No, it’s not that simple. Lots of people cancel their mammogram because they are scared. They may not like thinking of having private body parts examined. Think about walking into the hospital for the first time, knowing nothing. It will keep people from taking that first step.’* – Healthcare professional *‘We need to encourage people to talk about their stories about early diagnosis, cure and survivorship, because they have been on the journey.’* *‘It’s good to have young local people as translators in our healthcare practice, but older people might not feel comfortable having them to talk about their private body parts.’* – Healthcare professional *‘I think cancer is still so private for some people and families. For them, cancer may be perceived as unnatural, sad, and negative. It might be offensive for them if we keep asking for them to talk about cancer to others, when they are not yet ready for that.’* – Community member
Support for healthcare professionals	● Provide resources/education for healthcare professionals ● Provide cultural training for healthcare professionals ● Provide individualized services ● Hire healthcare ‘system navigator’	*‘[Healthcare system navigator] can help [healthcare professionals] communicate with [their] clients, help information flow, and support the client’s individual responsibility to seek out the information.’*

#### Recommendation 1: community outreach

Most participants agreed that community outreach promoting cancer screening should be a priority. A healthcare professional suggested that proactively reaching out would be more effective than passively waiting for individuals to participate in screening and educational opportunities. Community members also expressed concern about the limited awareness regarding available resources. While some informational resources are made available by community health centres, the type, source, location, and promotion of such resources may need to be re-evaluated.

Ensuring consistent messaging is disseminated to a broader segment of the community was another suggestion. Participants frequently discussed educating younger generations about cancer and cancer screening earlier, potentially in schools. A healthcare professional also expressed the importance of consistent awareness campaigns and outreach strategies that are carefully planned and implemented in a culturally safe manner. One community health promoter suggested incorporating awareness and educational activities within existing community events (e.g. community gatherings, drum circles, community feasts). Another participant, from a community cancer prevention programme, suggested utilizing a mobile diagnostic vehicle to improve screening service accessibility.

#### Recommendation 2: community collaboration

Community members emphasized the importance of community involvement in the planning and delivery of cancer screening services. Participants also noted that access to a wide support network in the community and healthcare system, such as community groups, Elders, a community health representative, and homecare visits, could be critical to advocating for increased screening.

Other participants asserted that increased collaboration among healthcare professionals was also needed. Medical information and expertise sharing among healthcare professionals could facilitate more effective and efficient cancer screening and follow-up services. Such communication may be difficult with transient healthcare professionals; it was suggested that working with community health promotion groups, like smoking prevention and cessation programmes, and establishing networks with other Northwest Territories communities would help transmit knowledge about successful cancer screening interventions. More broadly, agreements formalizing and standardizing cancer screening and care services between Northwest Territories and other provinces and territories (i.e. Alberta) could be an opportunity for jurisdictional collaboration.

#### Recommendation 3: sustainability

Providing sustainable and reliable services was indicated as critical to improving cancer screening utilization. Participants specifically suggested that the current government financial support policies should be reviewed, including those for medical travel. Increasing the sustainability of healthcare services at the community level, including through incentives to limit the turnover of healthcare professionals, could also strengthen screening services.

Educating and hiring local individuals as health advocates was also identified as a possible way to reduce turnover and increase trust within the community towards healthcare professionals. As well, when hired by major health centres, Northwest Territories physicians could be assigned to smaller surrounding communities. This practice would allow physicians to establish relationships and continuity of care through regular in-person visits and virtual support and provide relationship-based healthcare beyond the western standard. Healthcare professionals could also engage with the community and build relationships by attending community events, giving community presentations, and going on the local radio.

#### Recommendation 4: cultural safety

Community members suggested that establishing cultural safety would be an essential aspect of promoting screening services. The sharing circle methodology utilized for this project’s focus groups is as an example of a culturally safe approach. Participants suggested that sharing stories from cancer survivors through sharing circles would be a safe way to create openness around a topic needs more widely discussed. Healthcare professionals would also need to attend to the potentially complex cultural and historical root causes of reluctance towards utilizing cancer screening services. Participants further pointed out that ensuring cultural safety would not be simple; having young local individuals as translators could be culturally relevant, but not necessarily culturally appropriate, and more would be needed to ensure healthcare professionals offer a service that is culturally safe.

Community members also provided practical suggestions for increasing cultural safety, including hiring healthcare professionals and advocates familiar with local cultures and languages; the advocates could help explain procedures or tests in familiar terms, increasing patient understanding.

#### Recommendation 5: support for healthcare professionals

The final suggestion was to provide support, including education, to healthcare professionals, enabling effective implementation of the four approaches detailed above. Participants specifically recommended developing resources for healthcare professionals to use with community members, such as materials using local stories, local languages, images of community members, and which incorporate messages from community role models, such as Elders. Disseminating these materials using diverse media and venues for promotion, both new and traditional, would enable healthcare professionals to reach a wider audience. Participants also suggested that a sensitive language guideline for translating parts of the body could support communication between healthcare professionals and community members. One healthcare professional further recommended designating a *‘healthcare system navigator,’* a person in the community who guides individuals through the healthcare system. Cultural training and the importance of providing individualized services, such as gender-specific consultations, were also discussed.

## Discussion

This study outlines the unique insights and perspectives on cancer screening services held by healthcare professionals and Indigenous community members in Northwest Territories, a topic that has not been addressed in current literature. Participants identified both positive and negative influences on screening utilization, which were separated into four categories: socio-environmental, socio-cultural, socio-political, and personal. Participants also suggested five approaches for improving and supporting cancer screening services and utilization: community outreach, community collaboration, sustainability, cultural safety, and support for healthcare professionals. The findings of this study can help inform future interventions addressing the low rates of cancer screening utilization in Northwest Territories.

### Factors affecting the utilization of cancer screening services and suggestions for Northwest Territories

Factors that negatively impacted screening service utilization included personal preferences, limited healthcare resources, and cultural barriers (such as language). Factors identified in the present study are similar to those found in more urban settings [28] and by one study of in-depth interviews with 29 healthcare professionals involved in colorectal cancer screening across Northwest Territories [29]. Challenges regarding the use of medical terminology have also been noted elsewhere, regardless of whether English was the first language [30,31]. As well, while participants in our study recognized that ensuring cultural safety within screening services could be a complicated process, providing culturally safe cancer screening can positively influence utilization rates among Indigenous communities [32–35].

Several initiatives that could address the negative factors highlighted in this project have been implemented. Within a few months after the data collection, the Government of Northwest Territories initiated a new campaign called ‘Let’s Talk About Cancer’ to encourage conversations about cancer based on a series of regional sharing circles [36,37]. The campaign provides relevant cancer information (i.e. signs, symptoms, and stages) tailored for community members [10] and may have promoted the utilization of cancer screening services. Evidence presented by this work may guide future expansion of this initiative. Northwest Territories has started distributing a new colorectal screening tool [13] and the public trust and acceptance of this newer tool may need to be assessed. Northwest Territories has also implemented electronic medical record (EMR) keeping. The project participants, predominantly the healthcare professionals, expressed frustration with relying on physical charts and faxing referrals. EMRs may result in misfiling or healthcare professionals’ shortening or avoiding important discussions with patients [38]; however, EMRs have improved many areas of healthcare in Northwest Territories, including colorectal cancer screening [39].

Participants in our study believed building trust in the healthcare system will be essential for the sustainability of cancer screening services, especially for people with historical trauma or who may have had previously negative healthcare experiences. The historical context of residential schools and the resulting relationships between white authorities and Indigenous communities were identified as additional barriers to screening utilization within remote Indigenous communities that need to be addressed with community-based interventions. Specifically, this historical context was identified as a barrier to individuals taking active roles in decision-making around seeking screening services and follow-up care. Hesitancy from Indigenous community members may also be the result of systemic anti-Indigenous racism (including unconscious biases) [40,41]. This hesitancy could be perpetuated by a limited understanding of historical and cultural contexts from healthcare professionals at all levels and may result in distrust towards healthcare and healthcare professionals. A recent review of colorectal cancer screening in Indigenous communities found that distrust in western medicine was a significant barrier [42].

Cancer survivors could also play an important role as advocates in the community, opening the conversation and reducing fear around cancer by sharing personal stories and experiences of navigating the health system, particularly with medical travel. Patient advocates work with patients to facilitate patient-centred care by ensuring patients’ voices are heard during the healthcare journey, empowering and protecting patients, and creating space for empathy [43–46]. The benefits of patient advocates during cancer care have been highlighted in the literature [47,48]. Cancer screening used to be associated with fear, anxiety, and concerns about death [17,49]; however, many cancers are now seen as treatable, not as incurable diseases that result in death [30]. This shift in thinking is an important facilitator of screening service utilization; research on fear and behaviour shows that fear can trigger a behaviour change (i.e. being screened for cancer) [50], but if fear is overwhelming, fear-avoidance can occur (i.e. avoiding being screened) [51]. Fears around diagnoses, severe illness, and medication have been documented in other minorities living in rural areas [52]. Fear of death and other beliefs, such as that cancer is contagious, have also been noted as deterrents by healthcare professionals working with Indigenous communities [33]. Thus, as participants in this study identified, providing outreach services and education with culturally safe information is critical, a need which has been noted in studies exploring a Vietnamese American population [30].

As participants in this study shared, individuals may not be interested in screening services, particularly if no symptoms are experienced; this may be especially prevalent in the context of other priorities, such as food security and employment. Smith and colleagues found that 24% of cancelled colonoscopy appointments in Northwest Territories cited work or other commitments [53]. Participants in the present study indicated that, with some individuals, such attitudes may be difficult to change. For others, it may be less about attitude and more about social determinants of health (e.g. poverty, income inequalities, and racism). Nevertheless, the approaches recommended by participants may be a start to improving screening utilization.

Participants also noted that, beyond multi-format culturally safe information, there is a need for sustainable, sensitive, and supportive healthcare services and workers. Healthcare professionals need to be more aware of and sensitive to not only illnesses or diagnoses but to community members’ cultures and languages. Staff shortages, compounded by rapid staff turnover and transient healthcare professionals, may result in challenges for community members, such as difficulty building trusting relationships with healthcare professionals. Permanent healthcare professionals with community relationships are needed for such responsive, sustainable healthcare services. This need applies to services beyond cancer screening and is a priority for the provision of quality rural and remote healthcare [54]. In areas that rely on locums for healthcare professional services, additional steps need to be taken to ensure care sustainability, continuity, and cultural safety, and to establish trusting relationships with community members. Such educational and staffing recommendations have been made for the management of other diseases in Northwest Territories [55]. Experiential cultural training has been suggested in other areas of health as a way to improve confidence in and access to healthcare services within diverse populations [56]. Indigenous residents in remote communities in Northwest Territories are served by non-Indigenous healthcare professionals from the South. Arctic Indigenous communities may benefit from training healthcare professionals to maintain culturally safe approaches and perspectives that attend to the historical and social factors that have resulted in health discrepancies between Artic Indigenous communities and the rest of Canada. Participants also suggested that the use of a third party (e.g. local translator or healthcare system navigator) may be the preferred option to help both healthcare professionals and community members approach sensitive topics.

Overall, participants’ suggestions to improve cancer screening and utilization demonstrate the tight-knit nature of the communities and the importance of building multi-tiered relationships between healthcare professionals, communities, jurisdictions, and organizations. Further, some strategies for increasing colorectal cancer screening in Indigenous communities [57], such as outreach activities, culturally safe handout materials, and Indigenous representative advisory boards [57], have been used in seven provinces and Yukon. These efforts have been small and may be replicable, but would require scaling for larger outreach, which may present additional barriers [42].

### Strengths and limitations

To the authors’ knowledge, to date, this is the first study published specific to cancer screening within remote Indigenous communities in the Canadian Arctic. The large number of participants for this qualitative study came from a variety of backgrounds, including frontline healthcare professionals, community leadership, and community key informants. Trustworthiness of data analysis was enhanced by methodological triangulation (using data collected through multiple methods such as sharing circles, one-on-one semi-structured interviews, and field notes) and investigator triangulation (using multiple interviewers and analysts) [18,24]. The Community Advisory Board guided the culturally safe implementation of this community-based participatory project. The project team worked in a close partnership with the knowledge users to ensure the applicability and relevance of the findings in the context of Northwest Territories. The findings of this study were also utilized to inform the development of a video series about colorectal, breast, and cervical cancer screening services with Indigenous community members [58].

Some cautionary notes regarding the findings are worth mentioning. First of all, given the cultural diversity of the two included communities, the results of this study may not be generalizable to all Indigenous communities. The snowball sampling approach might have introduced a bias and limited representativeness of the participants; however, participants came from a wide variety of backgrounds. The sharing circles and interviews were conducted in English, and there may be some perspectives that could have been better illustrated in the local languages. The sessions were not audio-recorded and accuracy of data might have been impacted. To ensure as much accuracy as possible, both moderator and notetaker took detailed notes and debriefed right after each session. While gendered perspectives towards screening have been documented in other studies, with men tending to express more fear of potentially receiving positive results of cancer screening than women [59], the majority of participants in the present study identified as female. As such, the difference in genders was not prominent enough to allow for in-depth analysis on gender as a factor. Differences in opinions between healthcare professionals and community members should also be considered, especially when developing interventions and making policy changes. Although both groups identified barriers and provided suggestions for improvement, healthcare staff focused more on the physical environment and organizational logistics (i.e. faxing referrals), whereas community members focused on informational needs (whether it was accessible, understandable, and appropriate).

## Conclusions

This study contributes to the limited existing research available on cancer screening utilization in remote Indigenous communities; an understanding of the barriers to and facilitators of screening utilization can guide future educational and community-based interventions to improve screening utilization rates and support services in Indigenous communities. Some suggested approaches, such as the ‘Let’s Talk About Cancer’ campaign to reach out to communities and the development of the culturally safe cancer screening video series, have been implemented in Northwest Territories after data collection took place, highlighting the relevance and feasibility of the suggestions as concrete action steps. Further research is needed to identify and establish culturally safe strategies for delivering cancer and screening related information to Indigenous communities in the Arctic regions of Canada; this information must also harmonize culturally diverse perspectives on and approaches to cancer prevention. Tailoring the existing informational resources and healthcare system through community-led development programmes and strategies, increasing knowledge about cancer and cancer screening in a culturally safe way, and bringing together communities, healthcare professionals, organizations and systems into an extensive and in-depth system of cancer care can improve screening utilization rates.

## Supplementary Material

COREQ Checklist_251549442.pdf

## Data Availability

The datasets used and/or analysed during the current study are available from the corresponding author on reasonable request.
